# Draft genome analysis for *Enterobacter kobei*, a promising lead bioremediation bacterium

**DOI:** 10.3389/fbioe.2023.1335854

**Published:** 2024-01-08

**Authors:** Hossam S. El-Beltagi, Asmaa A. Halema, Zainab M. Almutairi, Hayfa Habes Almutairi, Nagwa I. Elarabi, Abdelhadi A. Abdelhadi, Ahmed R. Henawy, Heba A. R. Abdelhaleem

**Affiliations:** ^1^ Agricultural Biotechnology Department, College of Agriculture and Food Sciences, King Faisal University, Al-Ahsa, Saudi Arabi; ^2^ Biochemistry Department, Faculty of Agriculture, Cairo University, Giza, Egypt; ^3^ Genetics Department, Faculty of Agriculture, Cairo University, Giza, Egypt; ^4^ Biology Department, College of Science and Humanities in Al-Kharj, Prince Sattam Bin Abdulaziz University, Al-Kharj, Saudi Arabia; ^5^ Department of Chemistry, College of Science, King Faisal University, Al-Ahsa, Saudi Arabia; ^6^ Microbiology Department, Faculty of Agriculture, Cairo University, Giza, Egypt; ^7^ College of Biotechnology, Misr University for Science and Technology (MUST), 6th October City, Egypt

**Keywords:** bioremediation, genome analysis, heavy metal resistant bacteria, lead, qPCR, TEM

## Abstract

Lead pollution of the environment poses a major global threat to the ecosystem. Bacterial bioremediation offers a promising alternative to traditional methods for removing these pollutants, that are often hindered by various limitations. Our research focused on isolating lead-resistant bacteria from industrial wastewater generated by heavily lead-containing industries. Eight lead-resistant strains were successfully isolated, and subsequently identified through molecular analysis. Among these, *Enterobacter kobei* FACU6 emerged as a particularly promising candidate, demonstrating an efficient lead removal rate of 83.4% and a remarkable lead absorption capacity of 571.9 mg/g dry weight. Furthermore, *E. kobei* FACU6 displayed a remarkable a maximum tolerance concentration (MTC) for lead reaching 3,000 mg/L. To further investigate the morphological changes in *E. kobei* FACU6 in response to lead exposure, scanning electron microscopy (SEM) and transmission electron microscopy (TEM) were employed. These analyses revealed significant lead adsorption and intracellular accumulation in treated bacteria in contrast to the control bacterium. Whole-genome sequencing was performed to gain deeper insights into *E*. *kobei’s* lead resistance mechanisms. Structural annotation revealed a genome size of 4,856,454 bp, with a G + C content of 55.06%. The genome encodes 4,655 coding sequences (CDS), 75 tRNA genes, and 4 rRNA genes. Notably, genes associated with heavy metal resistance and their corresponding regulatory elements were identified within the genome. Furthermore, the expression levels of four specific heavy metal resistance genes were evaluated. Our findings revealed a statistically significant upregulation in gene expression under specific environmental conditions, including pH 7, temperature of 30°C, and high concentrations of heavy metals. The outstanding potential of *E. kobei* FACU6 as a source of diverse genes related to heavy metal resistance and plant growth promotion makes it a valuable candidate for developing safe and effective strategies for heavy metal disposal.

## 1 Introduction

While rapid technological advancements and industrialization contribute to fulfilling human needs and increasing their national income, they are considered the main source of hazardous waste material pollution, particularly heavy metals and other toxic compounds (Hubeny et al., 2021). Heavy metals, either essential (e.g., nickel (Ni), iron (Fe), manganese (Mn) and copper (Cu) or toxic [e.g., lead (Pb), mercury (Hg), arsenic (As), silver (Ag), cadmium (Cd) and chromium (Cr)], are generally harmful to living organisms even at low concentrations (Lemire et al., 2013). Likewise, it was reported that Pb, Hg, Cd, Cr, and As were considered the most toxic and carcinogenic metals (Mohamed et al., 2009; Masindi and Muedi, 2018). The toxicity of Pb leads to a variety of symptoms in the neurological system as well as the hematologic, hepatic, and renal systems (El-Beltagi and Mohamed, 2010; Flora et al., 2012). Pb also results in carcinogenesis, altered enzyme specificity, and disruption of cell membranes (Olaniran et al., 2013). Pb interacts with several proteins in renal cells, among them those that have been related to lead toxicity (Briffa et al., 2020).

Due to extensive industrial usage of metals, hazardous heavy metals have accumulated in the environment in significant amounts through effluents. These effluents are non-degradable, posing challenges for environmental disposal (Herawati et al., 2000). So, heavy metal accumulation negatively impacts the environment, especially when it enters the food chain (Tchounwou et al., 2012). The major sources of Pb in the environment include industrial processes, including the manufacturing of batteries, pigments, and metals, as well as the production of goods like lead arsenate pesticides or lead water pipes (Gadd, 2010). Therefore, it is crucial to investigate and identify an appropriate approach for treating this industrial wastewater before its discharge into the ecosystem, especially with the defects and problems that direct conventional treatment methods such as electrochemical removal, ion exchange and chemical precipitation (Gunatilake, 2015). Additionally, there is a need for effective and cheap technology to remove heavy metals using eco-friendly methods.

The use of biological agents as alternatives to chemical approaches for heavy metal removal has gained increasing attention. In this context, bioremediation can be regarded as a key, and effective method for ecological restoration and environmental cleanup (Abdelhaleem et al., 2019). Heavy metal-resistant bacteria have been isolated from different contaminated sites and studies on the interactions between these bacteria and heavy metals and other environmental pollutants to remove and reduce pollution hazards have been reported, such as lead (Mitra et al., 2021; Elarabi et al., 2023), chromium (Mohamed et al., 2020), cadmium (Saini and Dhania, 2020), arsenic (Balali-Mood et al., 2021), mercury (Kumari et al., 2020), nickel (Jan et al., 2019), herbicides (Elarabi et al., 2020) and polycyclic aromatic hydrocarbons (Abdelhaleem et al., 2019). There are five major mechanisms of heavy metal resistance (HMR) in the bacterial cell, including 1) extracellular barriers where the plasma membrane, cell wall, or capsule can prohibit the entry of metal ions into the cell; 2) metal ions active transport (efflux), which considers a method to export metal ions from the cytoplasm and includes three families of proteins [Cation Diffusion Facilitator (CDF), P-type ATPases and Resistance and Nodulation, Cell Division (RND)]; 3) extracellular sequestration, which contains insoluble chemicals or metal ions collected by biological components in the outer membrane; 4) intracellular sequestration, which prevents exposure to essential cellular components by accumulating metal ions in the cytoplasm in forms that are not bioavailable; and 5) reoxidation of metal ions (Choudhury and Srivastava, 2001).

Bacterial resistance mechanisms to heavy metals can be categorized into two main types: biochemical and molecular (Ma et al., 2016). The genetic determinants of resistance, encoded by genes located on bacterial chromosomes or plasmids, constitute the molecular mechanisms (von Rozycki and Nies, 2009). On the other hand, the bacterial resistance to heavy metals can be assessed by utilizing the bacteria’s Minimum Inhibitory Concentration (MIC) (Parameswari et al., 2010). *Enterobacter* plays great role in the bioremediation of multiple heavy metals (González-Henao and Ghneim-Herrera, 2021). It was reported that *Enterobacter cloacae* have high resistance levels to lead, cadmium, nickel and chromium (Pattnaik et al., 2020). *Enterobacter mori* and *Enterobacter aerogenes* were considered cadmium-resistant bacteria (Pramanik et al., 2018a). Otherwise, *E. kobei* was used as a lead-resistant bacterium (Abdollahi et al., 2020). Several recent advancements address the critical need to prevent the migration of heavy metals such as lead (Pb) and copper (Cu) into surrounding environments. These advancements include: Immobilization using loess and nanoscale zerovalent iron (nZVI). Wen et al. (2023) demonstrated the effectiveness of this method in immobilizing lead and reducing its mobility. Microbial Induced Carbonate Precipitation (MICP), this approach uses microbes to induce the precipitation of carbonates, which trap and immobilize heavy metals. Xue et al. (2023) and Xie et al. (2023a) further explored its application for remediating Pb-contaminated soil and water bodies. Biopolymer-assisted Enzyme-Induced Carbonate Precipitation (EICP), this technique utilizes enzymes and biopolymers to precipitate carbonates, effectively immobilizing heavy metals and preventing their migration. Xie et al. (2023b) highlighted its wide applicability for heavy metal remediation. Electrokinetic technology coupled with a biological permeable reactive barrier, this method uses electrical currents to drive the movement of contaminants towards a reactive barrier composed of biological materials, where heavy metals are removed and immobilized. Wang et al. (2023) studied its effectiveness in remediating heavy metal-contaminated loess. Combining these recent applications with the use of heavy metal-resistant bacteria has the potential to significantly improve the effectiveness of heavy metal removal.

The dramatic reduction in sequencing costs has made new genome sequencing technology increasingly available and affordable (Shendure and Ji, 2008). This has significantly enhanced our understanding of the biology of any organism, facilitated the identification of genome rearrangements, and simplified the investigation of novel genes during bacterial exposure to stress (Land et al., 2015). Also, genome analysis offers the opportunity to confirm the bacterial ability to resist unknown heavy metals (Huo et al., 2014). Additionally, this technique reflects the evolutionary dynamics of heavy metals and their relationship to environment (Ridge et al., 2008). Our investigation focused on isolating and characterizing a lead-resistant bacterium for bioremediation applications. By sequencing and analyzing its draft genome, we gained insights into the genetic mechanisms underlying its lead resistance. Moreover, the expression of four crucial genes associated with heavy metal resistance was studied.

## 2 Materials and methods

### 2.1 Sampling and evaluation of the samples’ physicochemical characteristics

Untreated industrial wastewater samples for bacterial isolation were collected from two sites in Sadat City, Menoufia Governorate, Egypt (30°26′30.6″N 30°38′24.7″E) in October 2019 (Supplementary Figure S1). The pH and electrical conductivity were measured following the method described by Elarabi et al. (2023) in triplicate. Using an atomic absorption spectrophotometer (Buck Model 210 VGP), the concentrations of As, Cd, Cr, Cu, Fe, Mn, Ni, Pb, and Zn ions were determined in the samples’ final solutions according to Page et al. (1983).

### 2.2 Pb-resistance bacterial isolation

The C_4_H_6_O_4_Pb, Na_2_HAsO_4_. 7H_2_O and K_2_Cr_2_O7 were purchased from Sigma and CdCo_3_ was purchased from Oxford laboratory. Pb-resistant bacteria were isolated and counted using the serial dilution method (Harley and Prescott, 2002). 0.1 mL of the diluted suspension was inoculated onto Luria Bertani (LB) media (10 g/L peptone, 5 g/L yeast extract, 10 g/L NaCl and 20 g/L agar: pH 7.00) supplemented with six different C_4_H_6_O_4_Pb concentrations (50, 100, 250, 500, 1,000, and 1,200 mg/L). Each isolate was assigned a lead resistance strain (LRS) name code.

### 2.3 Molecular identification

According to the manufacturer’s instructions, genomic DNA was purified from the bacterial isolates using the Simple™ Bacterial DNA Isolation Kit (Gene Direx, Inc., Cat. No. SN023-0100, Taiwan). Two universal primers targeting the *16S rRNA* gene, 27F, and 1492R, were used for molecular identification (Supplementary Table S1). The PCR reaction was carried out in a 50 μL total volume containing the following components: 50 ng/μL DNA, 2X One PCR™ Master Mix (Gene Direx, Cat. No. MB203-0100, Taiwan), 10 pmol of each primer, and the remainder of the volume to 50 μL with nuclease-free water. The PCR conditions were as follows: an initial denaturation step at 94°C for 5 min, followed by 40 cycles of denaturation at 94°C for 1 min, annealing at 58°C for 1 min, and extension at 72°C for 2 min. A final extension step at 72°C for 5 min was then performed. PCR products were purified using ExoSAP-IT™ PCR Product Cleanup Reagent (Applied Biosystems, United States, Cat. No. 78201). Purified DNA was sequenced by Sangon Biotech Co., Ltd, Macrogen, Korea. The *16S rRNA* gene sequences of the isolates and their closely related strains were aligned using ClustalOmega version 1.2.4 (Madeira et al., 2019). The *16S rRNA* gene sequence of each bacterial isolate was submitted to the National Center for Biotechnology Information (NCBI) GenBank database for comparison with published sequences. To further verify the identification, the sequences were also analyzed using the EzBioCloud Database (https://www.ezbiocloud.net/). Prior to phylogenetic analysis, the sequence alignment was trimmed using trimAl version 1.4.rev22 (Capella-Gutiérrez et al., 2009). Highly homologous sequences were selected and aligned using Clustal Omega. A phylogenetic tree was constructed using MEGA 11 software (Tamura et al., 2021) employing the Maximum Likelihood method under the Kimura 2-parameter model. Bootstrap analysis with 1,000 replications was performed to assess the confidence of the tree branches.

### 2.4 Estimation of the effect of Pb on cell survival, Pb biosorption efficiency and Pb uptake

The lead (Pb) resistance of bacterial isolates was measured using both the Minimum Inhibition Concentration (MIC) and the Maximum Tolerance Concentration (MTC) (Malik and Jaiswal, 2000). Bacterial isolates were cultured on LB agar plates supplemented with 10 different concentrations of lead acetate (ranging from 1,400 to 2,800 mg/L). Plates were incubated at 30°C for 15 days (Elarabi et al., 2023). After incubation, colony-forming units (CFUs) were counted to determine the survival of bacterial isolates. To assess the Pb removal ability of the isolates, LB broth supplemented with 2,600 mg/L lead acetate was inoculated with each isolate (this concentration was chosen as it is suitable and not stressful for all bacterial isolates). Pb absorption levels (mg/L), residual Pb ion concentration in supernatants, and Pb biosorption percentage were determined using inductively coupled plasma atomic emission spectroscopy (ICP-AES), following the method described by Shetty and Rajkumar (2009). Additionally, the survival and suppression percentages were calculated as described by Elarabi et al. (2023).

### 2.5 Microscopic analysis

Scanning electron microscopy (SEM) and transmission electron microscopy (TEM) analyses were conducted at the Cairo University Research Park (CURP), Faculty of Agriculture, Cairo University, Giza, Egypt, to determine potential Pb stress cells exhibit any structural changes and where metal accumulated. For SEM, a drop of the suspension from the bacterial culture, both with and without Pb, was placed on an aluminum stub and subjected to the procedures outlined by Khan et al. (2016). The gold film was then applied to samples using a sputter coater (Denton, Desk V HP) and viewed under a SEM (Nova NanoSEM 450). The TEM for the Pb-treated and untreated bacteria was carried out using a Tecnai G 20 transmission electron microscope (FEI, Limeil-Brevannes, France) SA 9900 at 200 kV (Burghardt and Droleskey, 2006). Images were captured using a high-resolution digital CCD camera and Olympus Soft Imaging System’s (Germany) iTEM image processing software.

### 2.6 Draft genome analysis

Following the manufacturer’s instructions, total DNA was extracted from *E. kobei* FACU6 using the QIAamp^®^ DNA Minikit (QIAGEN, Cat. No. 51304, Germany). The bacterium’s genome was then sequenced using the Illumina MiSeq™ platform (Illumina, United States) at the Genomics Research Program at the Children’s Cancer Hospital-Egypt 57357, Cairo, Egypt, employing NextGen High Throughput Sequencing technology. A conventional Illumina shotgun library, Nextera XT DNA Library Prep, was constructed and sequenced using the MiSeq platform. Paired-end sequencing generated 363,533 reads with an average length of 2 × 300 bp. The genome data was analyzed following the method described by Elarabi et al. (2023).

### 2.7 Determination of *E. kobei* FACU6 ability to resist other heavy metals

To confirm the ability of the *E. kobei* FACU6 strain to resist three different heavy metals (As, Cr, Cd), *E. kobei* FACU6 strain was grown on LB media supplemented with 1,500 mg/L Na_2_HAsO_4_. 7H_2_O (Pramanik et al., 2018a), 200 mg/L K_2_Cr _2_O_7_ (Iyer et al., 2004) or 1,000 mg/L CdCO_3_ (Pramanik et al., 2018b) to resist As, Cr and Cd respectively, in separate experiments and together in the same media. All cultures were incubated for 15 days at 30°C.

### 2.8 RNA extraction quantitative polymerase chain reaction (qPCR)

The *E. kobei* FACU6 strain, exhibiting the highest lead resistance, was used to evaluate the gene expression of various heavy metal (As, Cd, and Pb) resistance genes. The changes in mRNA levels of the *arsB, arsC, cadA, and pbrC* genes were assessed under different conditions of pH (5, 7, and 9), temperature (25, 30, and 42°C), and heavy metal concentrations (low, medium, and high) for each of Cd, As, and Pb. *E. kobei* FACU6 was cultured in LB medium supplemented with the three elements and incubated at 28°C for 3–15 days. The “low” concentration was 50 mg/L for all heavy metals. The “medium” concentrations were 500 mg/L for Cd, 1,250 mg/L for Pb, and 500 mg/L for As. The “high” concentrations were 1,000 mg/L for Cd, 2,500 mg/L for Pb, and 1,000 mg/L for As. In the combined three heavy metals experiment, 500 mg/L of each heavy metal was added to the medium. Total RNA was isolated from both metal-treated and untreated cells using the Simply™ Gene Direx Total RNA Isolation Kit (Cat. No. NA017-0100, Taiwan) according to the manufacturer’s instructions. Reverse transcription was performed using the Revert Aid First Strand cDNA Synthesis Kit (Thermo Scientific™, Cat. No. K1621, United States) with 250 ng RNA, following the manufacturer’s instructions. qPCR reactions were carried out in a 10 µL reaction volume containing 5 µL of 2X Maxima SYBR Green/ROX qPCR Master Mix (Thermo Scientific™, Cat. No. K0221, United States), 2 µL of 10X diluted cDNA, and 1 µL of each forward and reverse primer (Supplementary Table S1). The qPCR temperature program was as follows: 95°C for 2 min, followed by 40 cycles of 95°C for 15 s and 60°C for 60 s. The *16S rRNA* and *DNA gyrase* genes were used as reference genes.

### 2.9 Statistics analysis

GraphPad Prism 8 and R software were used to conduct one-way analysis of variance (ANOVA) and least significant difference (LSD) tests, respectively. The relative expression fold changes were calculated using the equation 2−ΔΔCT, as described by Livak and Schmittgen (2001). In addition, a two-way ANOVA test of multiple comparisons was performed in the gene expression experiment to measure whether the two datasets were significantly different (*p* > 0.005) using GraphPad Prism 8 software.

## 3 Results

### 3.1 Physicochemical analysis of collected samples

Electric conductivity (EC) and other physicochemical parameters were measured. As well as heavy metal contents were determined, as shown in Supplementary Table S2. The obtained samples had a slightly acidic, while the EC was low. In addition, the main hazardous metal concentrations in collected samples were measured. The findings showed that sample no. 1’s As, Cr, Cd, and Mn concentrations were found to be greater than United States Environmental Protection Agency (US EPA) screening limits, Conversely, Pb and Fe concentrations were higher in both samples (Supplementary Table S2) (US EPA, 2022). While the Pb concentrations in the two samples were similar, Cu and Zn were lower concentrations than the permissible concentration limit.

### 3.2 Selection of Pb resistant isolates

Twenty bacterial isolates were chosen due to their ability to survive on LB media supplemented with 1,200 mg/L of lead acetate. The MIC and MTC for these isolates were then determined by gradually increasing the Pb concentrations. The results showed that eight isolates named (LRS1, LRS2, LRS6, LRS9, LRS10, LRS15, LRS18, and LRS19) had a high MIC that reached 2,900 mg/L and a MTC of up to 2,800 mg/L (Supplementary Table S3). LRS19 exhibited the highest MIC and MTC (3,100 and 3,000 mg/L), respectively.

### 3.3 Molecular identification of the selected isolates

The *16S rRNA* genes of eight bacterial isolates were amplified, generating a 1,500 bp band that were subsequently purified and sequenced. The partial sequences of the *16S rRNA* gene were aligned and deposited to NCBI under accession numbers MT912742, MT912763, MW599729, MT912790, MT912758, MT912751, MW599731, and MW599732 as *K. quasipneumoniae* subsp. *ouasipneumoniae* strain FACU3 (98.71% similarity), *Klebsiella quasivariicola* strain FACU (99.22% similarity), *Citrobacter freundii*strain FACU 2 (99.52% similarity), *K. quasipneumoniae* subsp. *quasipneumoniae* strain FACU4 (98.55% similarity), *K. quasipneumoniae* subsp. *quasipneumoniae* strain FACU2 (99.66% similarity), *K. variicola* subsp. *variicola* strain FACU (99.73% similarity), *Enterobacter sichuanensis* strain FACU (99.9% similarity) and *Enterobacter kobei* strain FACU6 with (98.37% similarity), respectively. The phylogenetic tree was constructed as shown in Supplementary Figure S2. Also, the bacteria were deposited in the Culture Collection at Ain Shams University (CCASU WDCM1186, Cairo, Egypt) under designations CCASU-2022-26 to CCASU-2022-33 for *Klebsiella quasipneumoniae* subsp. *quasipneumoniae* FACU3, *K.quasivariicola* FACU, C. *freundii* FACU 2, *K. quasipneumoniae* subsp. *quasipneumoniae* FACU4, *K. quasipneumoniae* subsp. *Quasipneumoniae* FACU2, *Klebsiella variicola* subsp. *variicola* FACU, *E. sichuanensis* FACU and *E. kobei* FACU6, respectively.

### 3.4 Determination of the Pb effect on bacterial survival, biosorption efficiency and Pb uptake

The bacterial strain’s ability to resist Pb was evaluated through measurements of bacterial survival (%), biosorption efficiency (%) and lead uptake (mg/g dry weight). Significant differences were observed between the strains using a one-way ANOVA test and LSD tests. Additionally, the metal concentration in biomass (mg/g) was evaluated. The bacterial strains under investigation in this study have demonstrated a high efficiency in the uptake of heavy metals from the culture medium, with efficiencies ranging from 52.0% to 83.79%. This significant uptake has led to a substantial reduction in the residual concentration of heavy metals, as detailed in Supplementary Figure S3; Table 1. It was found that strain LRS19 (*E. kobei*), which can absorb 571.9 mg/g dry weight on strongly supplemented LB with 2,600 mg/L lead acetate had a highly significant percentage of bacterial survival under Pb stress (86.69%) and the best efficiency of biosorption (83.4%). The metal concentration in biomass was measured to be 1,024 mg/g in *E. kobei* while LRS15 (*K. variicola*) demonstrated the lowest significant percentage of bacterial survival under Pb stress (3.2%), with a biosorption efficiency of roughly 52.1 percent, capable of absorbing 87.6 mg/g dry weight and accumulate around 68 mg/g.

**TABLE 1:** Determination of the Pb effect on bacterial survival, biosorption efficiency, Pb uptake and metal concentration in the biomass.

Isolates code	Residual	Efficiency (%)	Biomass dry weight (g)	Metals uptake (mg/g dry weight)	Metals con. in biomass (mg/g)
LRS 1	484.4	81.3 a	0.114	464.5 b	587.8 b
LRS 2	1,213.1	53.1 d	0.428	80.6 e	221.8 f
LRS 6	773.9	70.1 c	0.224	204.8 d	336.6 e
LRS 9	615.9	76.2 b	0.133	371.1 c	532.3 c
LRS 10	779.2	69.9 c	0.261	173.4 d	413.9 d
LRS 15	1,239.1	52.1 d	0.385	87.6 e	68.9 h
LRS 18	1,170.7	54.8 d	0.348	102.1 e	86.4 g
LRS 19	429.6	83.4 a	0.094	571.9 a	1,024.1 a

The initial concentration was 2,588 mg/L.

### 3.5 Cellular morphological response to Pb stress by SEM and TEM

**FIGURE 1:**
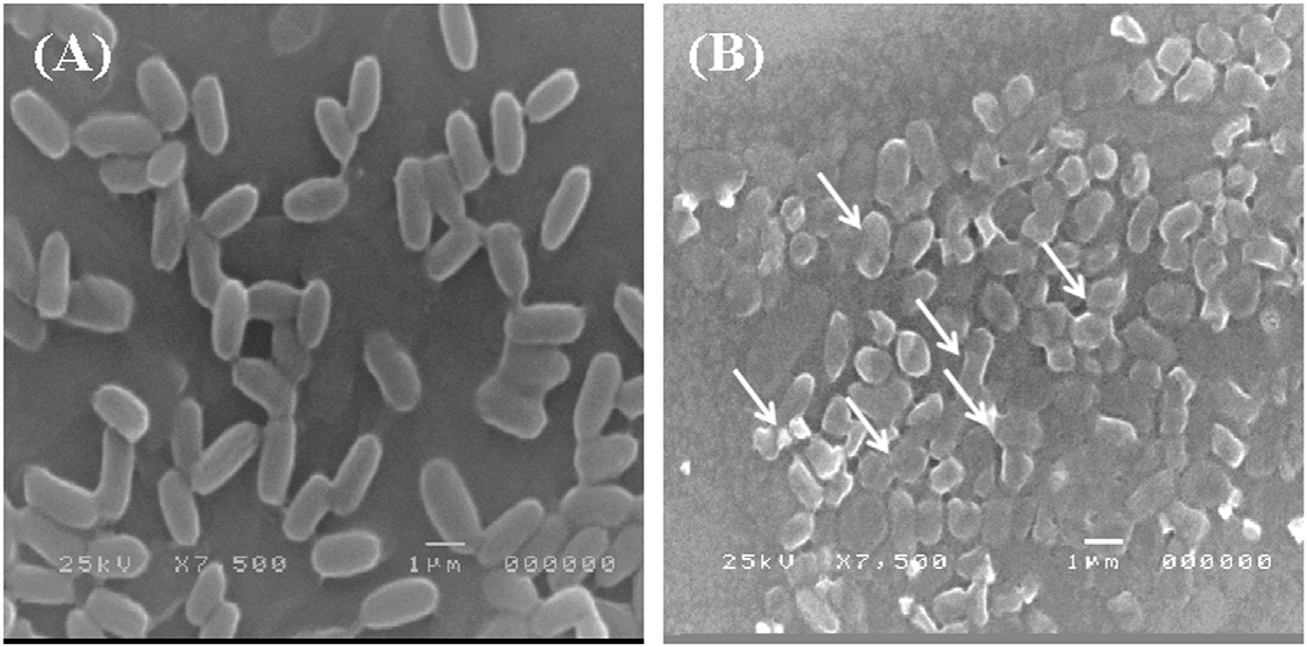
Scanning electron microscopic view of lead resistant *E. kobei*: **(A)** showing SEM pattern for absence of lead (control) and **(B)** for presence of lead. Arrows indicate the deformation in the bacterial cell compared with the untreated cells.

**FIGURE 2:**
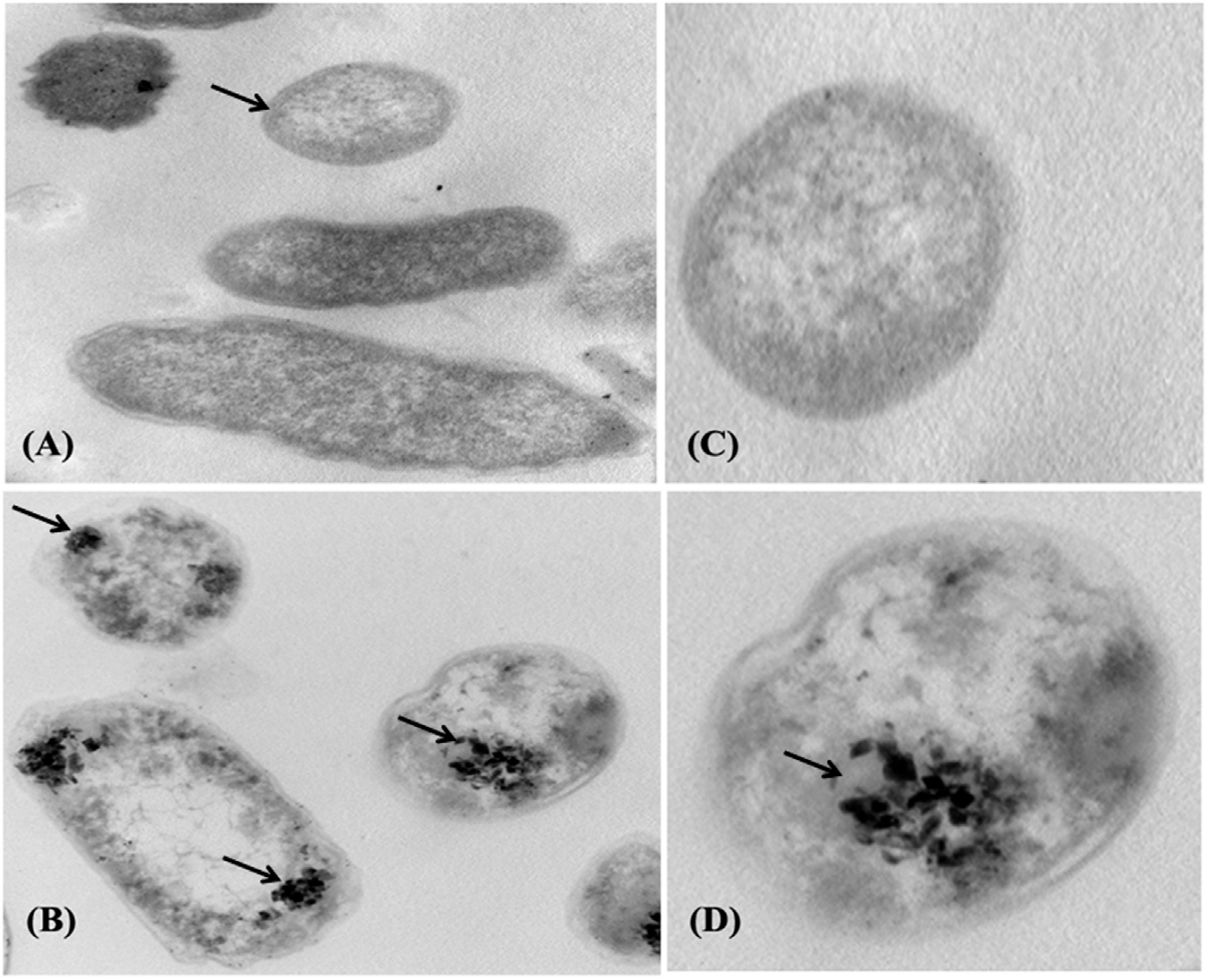
Transmission electron micrograph of the *E. kobei* strain cultured in liquid LB medium supplemented with 3000 mg/L lead acetate. **(A)** and **(C)** control - absence of metals; **(B)** and **(D)** with Pb. Arrows indicate locations of clearest contrasts of metal accumulation.

The adsorption of Pb onto the cell surface of *E. kobei* was observed using SEM. Comparing Pb-treated cells to the control, the SEM images revealed changes in cell morphology. In this study, The *E. kobei* cells in the control showed smooth exterior surfaces, were transparent, and had a uniform shape. All untreated bacteria displayed smooth, crystal-clear cell walls (Figure 1A), whereas the treated *E. kobei* cells were clumped or aggregated and had some shining particles on their surface (Figure 1B). Cells with clumped granular materials, deformed cells, protrusions that resembled configurations, and irregularly sized cells were all observed. After the biosorption of Pb, the bacterial cell wall was deformed. The SEM images demonstrated that Pb caused significant alterations in the shape of the *E. kobei* cell. By using TEM examination, it was possible to compare treated and untreated conditions’ Pb adsorption and intracellular accumulation. The biosorption ability of *E. kobei* was observed in the treated condition as numerous black dots, which were electron-dense materials or granules, were deposited mostly inside the cell or attached to the cell surface. In the images taken with TEM, an accumulation of Pb can be seen within the cell cytoplasm of *E. kobei* (Figures 2B, D) compared to untreated cells (Figures 2A, C).

### 3.6 Genome analysis

The genome analysis revealed 146 contigs with a total size of 4,856,454 bp and a G + C content of 55.06%, as shown in Table 2. The contigs’ N50 and L50 values were 84,952 bp and 18, respectively. Then 4655 CDS, 75 tRNAs and 4 rRNAs were identified using genome annotation. 497 putative proteins and 4,088 proteins with functional annotations were found among the protein-coding genes. The proteins with functional annotations included 1,258 proteins with Enzyme Commission (EC) numbers, 1,023 proteins with Gene Ontology (GO) annotations, and 890 proteins that were linked to Kyoto Encyclopaedia of Genes and Genomes (KEGG) pathways. According to the transporter categorization database (TCDB), 588 of the identified genes have similarities to recognized transporters, and 57 antibiotic-resistant genes according to CARD. DrugBank listed 310 gene targets for drugs.

**TABLE 2:** Genomic features of *E. kobei* FACU6 genome.

Features	Term
Contigs	146
GC %	55.06%
Contig L50	18
Genome Length	4856454
Contig N50	84952
CDS	4,655
tRNA	75
rRNA	4
Hypothetical proteins	497
functional Proteins	4,088
EC number Proteins	1,258
Go Proteins	1,023
Pathway Proteins	890
Antibiotic Resistance	57
Drug Target	310
Transporter	588
Bio sample	SAMN29718641
Bio product	PRJNA858462
Accession number	JANFOH000000000

**FIGURE 3:**
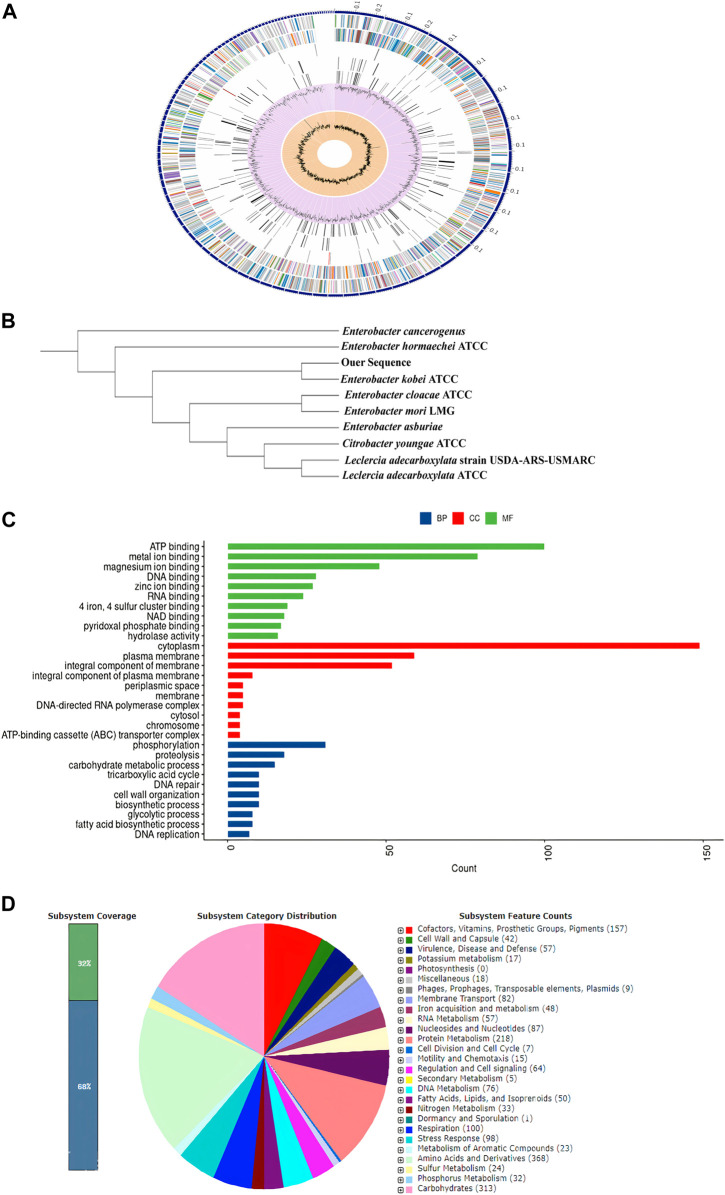
**(A)**: Using the PATRIC annotation tool, circular graphical representation of the *E. kobei* FACU6 genome’s distribution was created. The contigs are shown on the outer ring, followed by CDS on the forward and reverse strands, RNA genes, and CDS with homology to known antimicrobial resistance genes and known virulence factors. The GC content and GC skew are shown on the inner ring. **(B)**: Phylogenetic tree generated by IQ-TREE using the GTR1F1I1G4 model and visualized by iTol. **(C)**: Top 10 gene ontologies produced by UniprotR for biological process (BP), cellular component (CC), and molecular function. **(D)**: RAST was used to analyze subsystem coverage and category distribution of the entire *E. kobei* FACU 6 genome. The counts for each subsystem feature and the subsystem coverage are shown in the pie chart. Genes were displayed in brackets for each subsystem group.

Detailed properties and statistics of genome, in addition to the phylogeny relationship of *E. kobei* FACU6 between *Enterobacter* genus, are summarized in Table 3; Figures 3A, B. Together with the top 10 gene ontologies and gene count for biological processes (BP), cellular components (CC), and molecular functions, as depicted in Figure 3C. In addition, three plasmids having IncFII, Col and IncFIB replicons with identities of about 96.92%, 96.12%, and 98.93%, respectively, when comparing to the databases in PlasmidFinder, on three different contigs, were identified in this genome. The three plasmids were also confirmed using the Abricate database. Furthermore, Figure 3D shows the subsystem coverage and category distribution of the entire *E. kobei* FACU6 genome. The results demonstrated that this genome contains approximately 27 subsystems. One of these subsystems was the virulence, disease, and defense subsystem, which contained approximately 57 genes. Twenty-nine heavy metal genes were annotated in the *E. kobei* FACU 6 genome.

**TABLE 3:** Heavy metal resistance genes in *E. kobei* FACU6.

Genes	Function	Length	Heavy metal resistance	Locus tag
ZntA*	Cadmium, lead/Zinc transporting P-type ATPase	2,187	Cd, Zn, Pb	NM529_02811
ZntR*	HTH-type transcriptional regulator	369	Cd, Zn, Pb	NM529_01330
		423		NM529_01330
zntB*	Zinc transport	984	Zn	NM529_01622
*chrR#*	Quinone reductase	567	Cr	NM529_03023
*arsC#*	Arsenate reductase	432	As	NM529_00630
*ArsB#*	Arsenical pump membrane	1,290	As	NM529_00631
rcnA*	Nickel/cobalt efflux system	876	Co,Ni	NM529_00192
rcnR*	Transcriptional repressor	312	Co, Ni	NM529_00193
rcnB*	Cobalt/nickel homeostasis	333	Co, Ni	NM529_02252
hoxN*	Nickel transport	1,023	Ni	NM529_04404
zitB*	Zinc transporter	939	Zn	NM529_00523
znuA*	Zinc uptake	681	Zn	NM529_01941
znuC*	Zinc import ATP-binding protein	756	Zn	NM529_01943
znuB*	zinc uptake system	786	Zn	NM529_01944
yeiR*	Zinc-binding GTPase	987	Zn	NM529_02305
Zur*	Zinc uptake regulation	513	Zn	NM529_03801
ftsH*	ATP-dependent zinc metalloprotease	1935	Zn	NM529_03964
zupT*	Zinc transporter	774	Zn	NM529_04111
*copA#*	Copper-exporting P-type ATPase	2,499	Cu	NM529_00223
*pcoE#*	putative copper-binding	312	Cu	NM529_01486
cutC*	Copper homeostasis	744	Cu	NM529_01956
cueO*	Blue copper oxidase	1,599	Cu	NM529_03698
cueR*	transcriptional regulator	414	Cu	NM529_00224
*cusA#*	Cation efflux	3,135	Ag, Cu	NM529_01487
*cusB#*	Cation efflux	1,251	Ag, Cu	NM529_01488
*cusF#*	Cation efflux	342	Ag, Cu	NM529_01489
*cusC#*	Cation efflux	1,386	Ag, Cu	NM529_01490
*cusR#*	Cation efflux	714	Ag, Cu	NM529_01491
*cusS#*	Sensor histidine kinase	1,461	Ag, Cu	NM529_01492

Several genes were found in multiple copies in different locations on every cell. Figures represent pertinent contigs; The Prokka annotation provided the locus tags. #Plasmid-based genes and *Chromosome-based genes

Some of those genes, including Pb, Cd, Cr, As, Ni, Co, Cu, and silver (Ag), the genes identified were discovered to be implicated in heavy metal resistance genes. In addition, resistance, including those associated with lead (Pb), cadmium (Cd), chromium (Cr), arsenic (As), nickel (Ni), cobalt (Co), copper (Cu), and silver (Ag).

**FIGURE 4:**
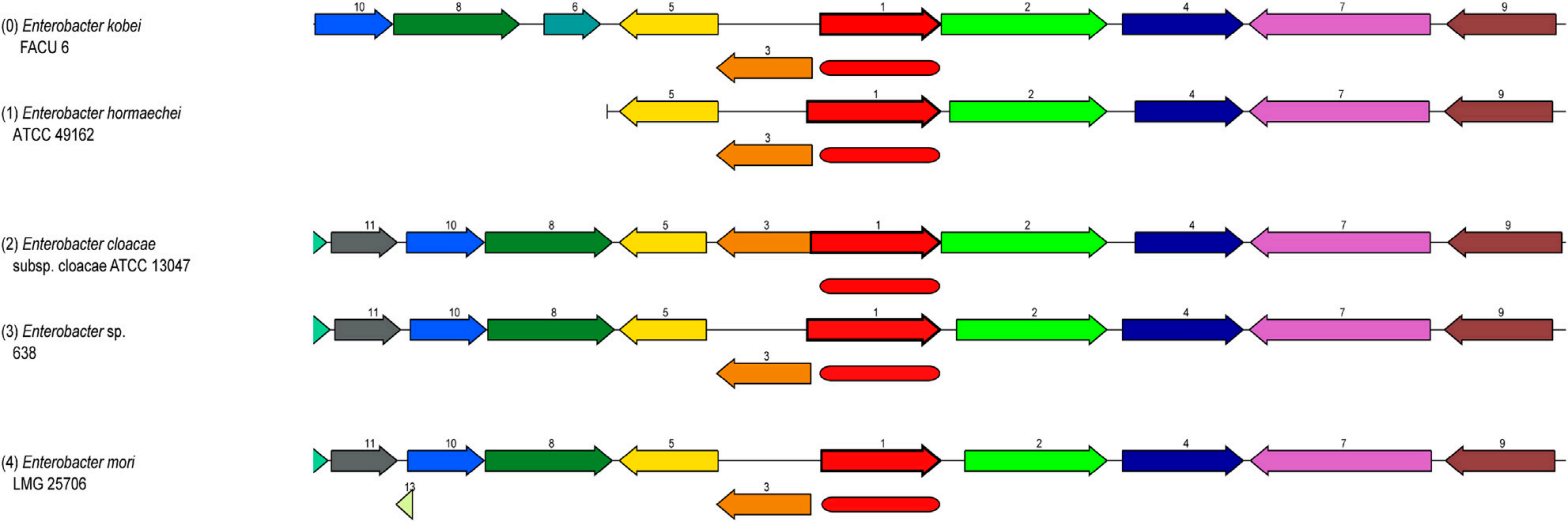
Zinc resistance gene cluster: Four closely related organisms’ chromosome regions were compared with the region of the focal gene (top). The focus gene, which is red and has the number 1, is shown in the graphic. The same number and color are used to identify groups of genes with identical sequences (*1-Zinc ABC transporter, substrate-binding protein ZnuA, 2-Murein DD-endopeptidase MepM, 3-Zinc ABC transporter, ATP-binding protein ZnuC, etc.). Myristoyltransferase for 4-Lipid A biosynthesis, ZnuB, a putative protein, a 5-Zinc ABC transporter and kinase 7-pyruvate, RuvB, an ATP-dependent DNA helicase for the Holiday junction, HexR, the RpiR family, and 11-Crossover junction endodeoxy ribonuclease RuvCand 10-Holliday junction ATP-dependent DNA helicase RuvA*).

Some of these genes, those related to resistance against heavy metals such asPb, Cd, Cr, As, Ni, Co, Cu, and silver (Ag), to be heavy metal resistance. In addition, some genes were found in complete clusters, such as the zinc resistance gene cluster (Figure 4). The *E. kobei* FACU 6 zinc resistance gene cluster’s chromosomal region was compared to four other bacteria (*E. cloacae* subsp. *cloacae* ATCC 13047, *Enterobacter* sp 838, *E. hormaechei* ATCC 49162 and *E. mori* LMG 2506). The results displayed complete similarity in the zinc resistance gene cluster between these species. These results indicated the ability of *E. kobei* FACU 6 to resist various heavy metals, including Zn, Cr and Cd. Moreover, this genome harbored about 65 plant growth-promoting genes (PGPGs) like genes involved in indole acetic acid (IAA) production, acetoin and butanediol synthesis, trehalose metabolism, phosphate solubilization, chitinase, phenazine, 4-hydroxybenzoate, cold shock proteins, heat shock proteins, glycine-betaine, H_2_S, peroxidases, catalases, superoxide and siderophore production (Table 4). The majority of the AMR genes were engaged in conferring resistance via efflux pumps or changing antibiotic targets, as shown by Table 5, which summarizes the identified AMR genes in this genome and their corresponding mechanisms. Supplementary Table S4 shows the results of the RGI tool’s analysis of the *E. kobei* FACU6 resistome, which included 1 perfect hit and 19 strict hits. The strain is expected to be resistant to carbapenem, cephalosporin, cephamycin, and penam due to the presence of several resistant genes.

**TABLE 4:** List of *E. kobei* FACU6 genes that promote plant growth.

Genes	Plant growth promotion properties
*pstS, A, C, B*glucose dehydrogenase	Phosphate solubilization
*budA, B, poxB*	Acetoin and butanediol
*ipdC*	IAA
*chiA*	Chitinase
*phzF*	Phenazine
*treS, Y, C, B, otsA, B*	Trehalose
*ubiA*	4-hydroxybenzoate
*hspQ, ibpA,R, hslJ, ibpB*	Heat shock proteins
*CysZ, M, G, W, A, L, P, Q, E, E,I, T, E,H, N,K,D*	H_2_S
*CpsB, A, E, C, D,*ydfK	Cold shock proteins
*osmC, oxyR, srpA, cpo, efeB, butE, tpx*, *yfeX*	Peroxidases
*proX, W, V*	Glycine-betaine
*kat G,E*	Catalases
*yfiZ, fhuABCDF, yusV*	Siderophore
*sodA*	Superoxide dismutase
*nirD, fdnGHI, narGHIJKLQUVWXYZ, fnr*	Denitrification

**TABLE 5:** The genome’s annotated AMR genes and associated AMR mechanisms.

Genes	ABR mechanism
*KatG*	Activationantibiotic
*ACT/MIR* family, *CatA* family	Inactivation antibiotic
*MarR, MarA, MarB*	Resistance antibiotic
*Alr, dxr, Ddl, S10p, EF-Tu, EF-G, MurA, folA, folP, gyrA, Dfr, inhA, gyrB, Iso-tRNA, kasA, fabI, rho, rpoC, rpoB, S12p*	Antibiotic target
*BcrC*	Antibiotic target
*MdfA/Cmr, AcrEF-TolC, AcrAB-TolC, AcrAD-TolC, AcrZ, EmrD, MacA, SugE, MacB, EmrAB-TolC, MdtL,MdtABC-TolC, TolC/OpmH*	Efflux pump
*GdpD, PgsA*	Antibiotic resistance
*OccD6/OprQ*	Antibiotic resistance
*H-NS, AcrAB-TolC, OxyR, EmrAB-TolC*	Antibiotic resistance

### 3.7 Evaluation of *E. kobei* FACU6 ability to resist other heavy metals

**FIGURE 5:**
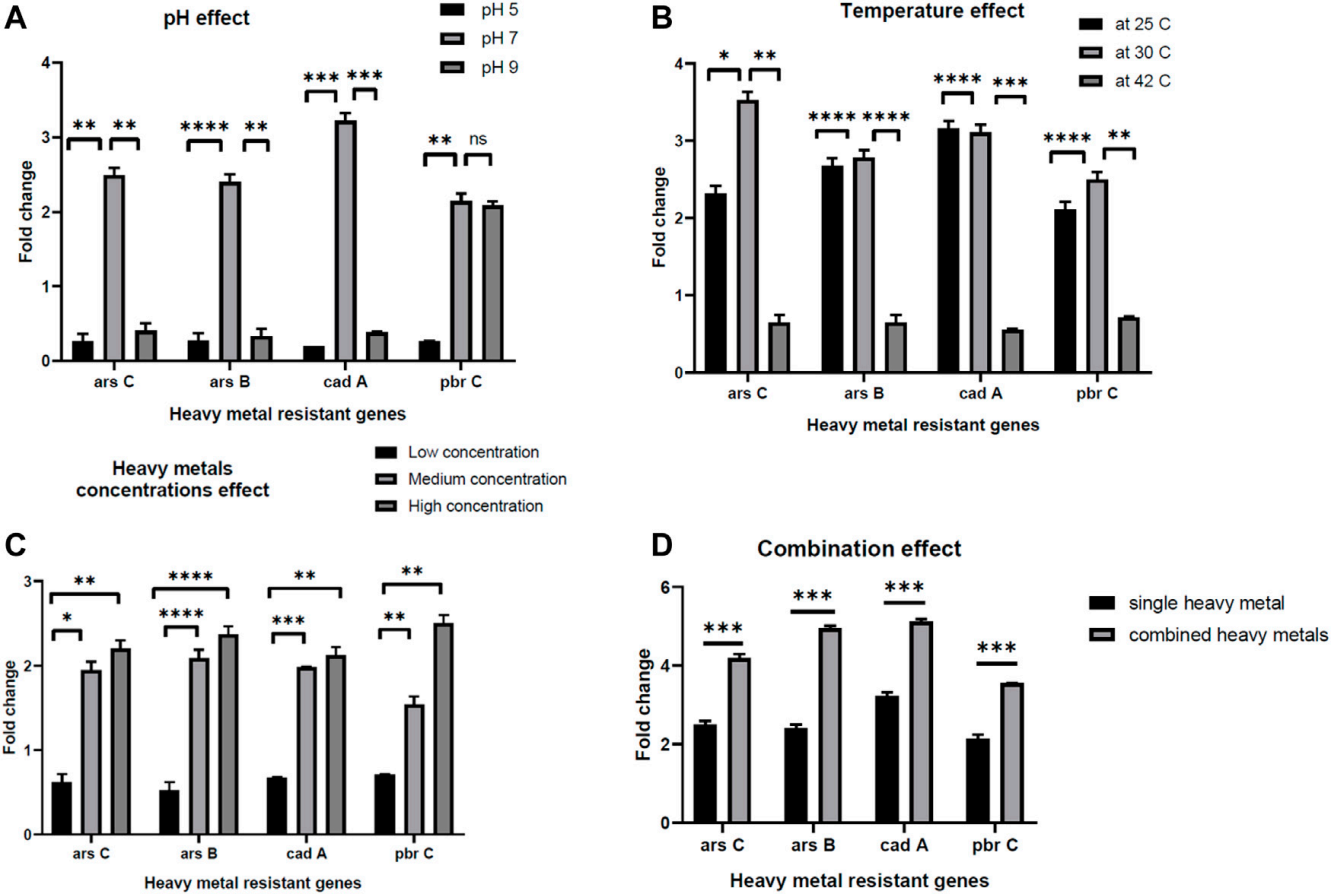
The effect of pH, temperature, heavy metal concentrations and combination effect **(A–D)** on gene expression of *arsC, arsB, cadA, pbrC* genes in *E. kobei* FACU6.

The ability of the *E. kobei* FACU6 strain to resist additional heavy metals was assessed. It was found that *E. kobei* FACU6 could grow in media supplemented with As, Cr, or Cd in addition to Pb. The results showed that *E. kobei* FACU6 could tolerate 1,500 mg/L, 200 mg/L and 1,000 mg/L of As, Cr, and Cd, respectively. In addition, *E. kobei* FACU6 was able to grow in LB media supplemented with the four heavy metals. To confirm the ability of *E. kobei* FACU6 to resist different heavy metals, qPCR was utilized to determine messenger RNA expression levels of two As resistance genes (*arsB, arsC*), one Cd resistance gene (*cadA*) and the *pbrC* gene for Pb resistance under different conditions. There was a highly significant difference between pH 7, 5, and 9. The expression of *arsB, arsC, cadA,* and *pbrC* genes in *E. kobei* FACU6 increased significantly (*p* 0.05) under pH 7, with a fold change of 2–3 times (Figure 5A). For the different temperatures tested, all genes were downregulated at 42°C, while their expression at 25°C and 30°C was upregulated with fold changes ranging from 2 to 3.5 times (Figure 5B). Furthermore, the effect of heavy metal concentration on gene expression of these genes was determined individually and combined in LB media. Gene expression has a positive relationship with the increase in heavy metal concentrations, with highly significant differences between them (Figures 5C, D). The previous results concluded that *E. kobei* FACU6 is considered a multi-heavy metal resistant isolate due to its ability to resist Pb, Ar, Cr, and Cd with high tolerance concentrations.

## 4 Discussion

Isolating bacteria resistant to heavy metals from contaminated sites is a critical step in the bioremediation process. In this study, the industrial zone of Sadat City was chosen for bacterial isolation since it is one of the biggest industrial zones in Egypt. In this region, it is common to find industries in batteries, plumbing, painting, wiring, electric cables, raw material mining, and ceramic glazing. The physicochemical properties of both soil samples were analyzed. The pH was slightly acidic, which was reflected in the EC, which had low conductivity. The lead industries’ byproducts may cause low pH and EC (Nirbhavane et al., 2017). Also, it was observed that sample no. 1 was more contaminated than sample no. 2, with high levels of several heavy metals that exceeded the allowed concentration limit (US EPA, 2022). According to their MICs, eight bacterial isolates were selected. These isolates were molecularly identified and submitted to GenBank. Five strains belonged to the genus *Klebsiella*, two to the genus *Enterobacter*, and one to the genus *Citrobacter*. According to reports, the *Enterobacter* and *Klebsiella* species are useful bacteria in the treatment of antibiotic and heavy metal resistance (Wang et al., 2018). *Citrobacter freundii* has also been applied to obtain biosurfactants and remove heavy metals (Gomaa and El-Meihy, 2019).

In addition, significant variations in bacterial survival, biosorption efficiency, and lead uptake were observed among the strains. The results indicated that the significantly low percentages of bacterial survival, biosorption efficiency, and lead uptake may be attributed to the strains reaching the level of toxicity, and thus lead pressure influenced cell survival. If toxic pressure is insufficient to kill the organisms, it can increase cell membrane permeability, subsequently increasing toxic ions’ uptake (Odokuma and Akponah, 2010). The results showed that the *E. kobei* (LRS 19) strain displayed the highest level of Pb resistance, whereas the *K. variicola* (LRS 15) strain exhibited the lowest level. While the MIC of the promising strain *E. kobei* FACU6 reaches 3,000 mg/L with efficiency (83.4%), Abdollahi et al. (2020) tested the ability of *E. kobei* isolated from soil collected from sites near a lead-zinc factory in Iran to resist lead and found that it had an accumulation capacity of 25% in addition to its ability to resist cadmium. Moreover, the binding of lead ions on bacterial cell surface was also observed using SEM. The cellular surface of a bacterial cell has been considered the most potent sorbent that adsorbs metal ions and minimizes their harmful effects (Kang et al., 2020). In Gram-negative bacteria, the cells have negatively charged lipopolysaccharides in their cell walls provide the ability to bind to cationic metal ions and give them a metal binding feature (Khan et al., 2015). Since metals are not biodegradable and cannot be broken down. Organisms can detoxify metal ions by enclosing the active component in a protein or storing them insoluble in intracellular granules. Furthermore, in *E. kobei* FACU6, the TEM data showed a clear distinction between control and Pb-treated cells in terms of the size and thickness of the cell wall. On the cell membrane and in the cytoplasm, black granules were observed. Generally, heavy metals inevitably enter the cells, accompanied by nutrient uptake by microorganisms (Cuypers et al., 2010). Consequently, accumulated heavy metals may harm bacterial cells (Cuypers et al., 2010). To ensure their survival, normal growth, and metabolism, bacterial strains employ various defense mechanisms, including extracellular sequestration, biosorption, precipitation, efflux, intracellular bioaccumulation, and changes in cell morphology (Naik and Dubey, 2013). The first structure in a cell to interact with metal ions is the cell wall, which has carboxyl, phosphate, sulfhydryl carbonyl or hydroxyl groups as functional groups. These groups play a significant and fundamental role in the biosorption of metal ions (Khan et al., 2022). They negatively charge the cell wall and produce insoluble compounds when they bind to lead ions. The cell wall, composed of organic macromolecules including polypeptides, polysaccharides, and proteins, could also adsorb Pb by electrostatic forces like Van der Waal’s forces, covalent or ionic bonds (Qiao et al., 2019). Thus, this could explain the molecular mechanisms of lead resistance in *E. kobei* FACU6.

Bacteria possess high surface-to-volume ratios and active sites in their cell walls, which serve as potential metal accumulators (Sah et al., 2022). This characteristic likely contributes to the observed high efficiency of heavy metal uptake. However, an interesting observation was made regarding the metal concentration within the bacterial biomasses. Despite the high uptake, the concentration within the biomasses was found to be significantly lower or moderated for all strains investigated. For instance, the metal concentration within the LRS1 strain biomass was recorded at 9.23 mg/g. This discrepancy could be attributed to this discrepancy, including adsorption of metals onto the bacterial cell surface or extracellular precipitation (Maity et al., 2023). SEM result provided visual evidence supporting these mechanisms. Furthermore, it was found that LRS19 bacterial strain has ability to reduce heavy metal concentrations in their environment through intracellular accumulation, which clarifies the high metal concentration in its biomass (1,024 mg/g). However, it is important to note that the relative contribution of each mechanism can vary depending on specific environmental conditions and types of heavy metals present (Ayele and Godeto, 2021). To further investigate the Pb-resistant genes and removal mechanism and metabolic pathways, whole-genome sequencing (WGS) was performed for *E. kobei* FACU6. The genome was larger than previously reported *E. kobei* M4-VN that was previously disclosed (Thanh et al., 2020). *E. kobei* FACU6 contained a high *tRNA* gene content. The significant *tRNA* gene content may be a result of the cell’s ability to regulate gene expression in microorganisms in habitats with a wide range of conditions (Rodríguez-Rojas et al., 2016). In addition, three plasmids, IncFII, Col and IncFIB replicons, were found in the *E. kobei* genome. Referring to the minimal genome sequence information, revealed that the genome possesses an extensive number of heavy metal resistance genes and gene clusters, as shown in Table 4. Compared to metal-resistant bacterium *E. kobei* CPE isolated from wastewater in the UK, which carried arsenic, mercury, and tellurium resistance genes (Ludden et al., 2017), the draft genome of *E. kobei* FACU6 revealed the presence of *arsBC* pair genes encoding for the proteins *arsA* and *arsB*, which participate in arsenic resistance but didn’t have any specific resistance genes for tellurium and mercury. The genome also contains clusters and system genes involved in the transport and resistance to metals like cadmium, zinc, nickel, and cobalt. *E. kobei* FACU6 lacks the Pbr, Czc, and Cad operons, although earlier studies suggested that heavy metals (Zn, Pb, Co, and Cd) translocating P-type ATPase genes can also function in lead resistance. The *E. kobei* FACU6 genome contained the *zntA* gene, which encodes a zinc/cadmium/lead-transporting P-type ATPase (Hui et al., 2022). Not only zinc but also lead and cadmium greatly increased the expression of *zntA*, in a process uncontrolled and mediated through *zntR* (Binet and Poole, 2000). *zntR* and *zntA* genes were located at different locations on the chromosome (Baya et al., 2021). This confirms our findings about the lead resistance of *E. kobei* FACU6. Lead-resistant bacteria can use a variety of methods, including biosorption, efflux mechanisms, induced precipitation, extracellular sequestration, and intracellular lead bioaccumulation, in Pb bioremediation process (Jarosławiecka and Piotrowska-Seget, 2014). When Pb is present, an influx transporter, such as P-type ATPases, can move and deposit Pb into the periplasmic region of the bacterial cell (Huang et al., 2018). The resistance gene *Pbr* functions as efflux pump to transport and immobilize Pb on cell surface in insoluble forms as Pb concentration rises, minimizing the toxicity caused by Pb (Jarosławiecka and Piotrowska-Seget, 2014). Microorganisms can transform harmful metal ions into insoluble complexes like phosphate, carbonate and sulfate during precipitation, lowering their concentration and, as a result, the toxicity of contaminated areas (Naik and Dubey, 2013). *ZntA* stimulates metal translocation across inner membrane, and molecule is transported to periplasm from the cytoplasm (Klein and Lewinson, 2011). While González Henao and Ghneim-Herrera (2021) examined the absence of *pbr* genes in several genera, including *Bacillus, Pseudomonas, Klebsiella, Microbacterium, Agrobacterium, Rhodococcus, and Enterobacter,* Hynninen et al. (2009) suggested that lead detoxification by ATPases and phosphatases was a common mechanism for lead tolerance in these microorganisms. Therefore, more research is needed to understand these metal resistance genes and systems in many bacterial species. *ZntB*, which serves as a zinc efflux method, was present in *E. kobei* FACU6 (Worlock and Smith, 2002). *ZitB* is crucial for zinc homeostasis at low zinc concentrations, but *znt A* is crucial at high zinc concentrations (Grass et al., 2002). *Zur* gene and *znuABC* operon were identified in *E. kobei* FACU6, and it was reported that the *zur* gene product reportedly responds to intracellular zinc concentration to regulate the zinc transporter expressed by *znuABC* gene cluster (Patzer and Hantke, 2000). In addition to transporting cadmium and copper ions, *ZupT* also mediates the absorption of zinc (Grass et al., 2002). *YieR* participates in metal hemeostasis (Blaby-Haas et al., 2012). Previous research has shown that *E. kobei* possesses several zinc resistance gene systems and gene clusters.

The nickel-cobalt efflux system’s rcn-operon (*rcnA, R,* and *B*) was found in *E. kobei* (Blaha et al., 2011). *Hox N* gene, which is characterized as a high-affinity nickel transport protein facilitating nickel transport, was also present in *E. kobei* (Wolfram et al., 1995). Our results showed that *E. kobei* FACU6 additionally possessed various arsenic and chromium resistance genes. The arsenate reductase (*arsC*) protein is necessary for arsenic resistance. Arsenate reductase, which is encoded by the existing operon (*arsRBC*), was able to convert arsenate into arsenite, and the integral membrane protein arsB encoded the remaining steps of the process. This prevented arsenic buildup by expelling it out of the cytoplasm (Yang et al., 2012). Notably, the quinone reductase gene (*chrR*) contributes to chromate bioremediation by decreasing chromate (Paul et al., 2020). In addition to the *cus ABCFRS* operon, which is involved in the silver efflux system, gene systems or gene clusters were encoded for copper hemeostasis, such as *copA, pcoE, cutC, cueO*, and *R* (Gudipaty et al., 2012). Moreover, *E. kobei* was considered a multi-heavy metal resistant bacteria and it harbors antibiotic resistant genes. Their AMR mechanisms revealed that most genes contributed to resistance through efflux pumps and altered antibiotic targets. Multiple resistant genes were expected to confer resistance to carbapenem, cephalosporin, cephamycin, and penam. This has been verified by numerous studies showing that heavy metal induction can affect both antibiotic-resistance genes and heavy metal-resistance genes in bacteria. These studies also demonstrated that heavy metal contamination of ecosystem can affect antibiotic resistance genes (Chen et al., 2019). It was important to note that genes that support plant growth in *E. kobei* can help increase nutrient availability, resistance to oxidative stress, and suppression of biotic and abiotic stress. *E. kobei* genome contains the indole pyruvate decarboxylase (*ipd*) gene, which converts tryptophan to IAA (Straub et al., 2013). Moreover, the tryptophan biosynthesis-related *trp* cluster (*trpA, B, C, R,* and *S*) genes were identified. These genes may contribute to the production of tryptophan, a building block for the biosynthesis of the IAA hormones that promote plant development (Duca et al., 2014). Genes for glucose dehydrogenase activity and the phosphate-specific transport system (*pst*) operon, which are involved in the solubilization of mineral phosphates in soil, were found in the genome of *E. kobei* (Brito et al., 2020). Moreover, *E. kobei* had genes involved in hydrogen sulfide (H_2_S) biosynthesis, which contribute to seed germination and accelerate plant growth (Dooley et al., 2013). Genes involved in the production of 2, 3-butanediol and acetoin, which are known to promote plant growth, were also found in *E. kobei*, including *budA, B, C,* and *poxB* (Ryu et al., 2003). Furthermore, many genes encoding catalases, peroxidases, and superoxide dismutase, all of which reduce oxidative stress in plants, were also found in the *E. kobei* genome (Rai et al., 2013). Additionally, the *E. kobei* genome contained the genes *phzF* and *ubiA*, which were responsible for the synthesis of phenazine and 4-hydroxybenzoate, respectively. These genes also supported plant growth by reducing osmotic stress and promoting plant defense and communication, respectively (Yuan et al., 2020). In addition, genes that allow bacteria to resist abiotic stress such as heat shock, cold shock, trehalose synthesis, and glycine-betaine production were discovered. This bacterium can be employed in biocontrol because the *chiA* gene, which encodes chitinase synthesis and is important for the nutritional cycling of chitin, was discovered (Veliz et al., 2017). *E. kobei* genome contained *yfiZ, yusV,* and *fhu* clusters, which participate in iron acquisition (Veliz et al., 2017). According to Papaspyrou et al. (2014), denitrification had ecological importance, and the findings revealed the genes in the control of nitrate reduction to ammonia. Taking the aforementioned results into consideration, it can be concluded that *E. kobei* FACU6 possesses not only multi-level heavy metal resistance but also the potential to promote plant growth.

To confirm the *E. Kobei* FACU6 genome annotation results, the ability of the bacteria to grow in LB media containing a mixture of heavy metals was assessed, along with the gene expression of some genes involved in heavy metal resistance (*arsB, arsC, cadA,* and *pbrC*). In the gene expression experiment, several factors were used to assess the heavy metal resistance genes, including pH, temperature, heavy metal concentration, and combined heavy metals in LB media. A highly significant difference was observed between pH 7, 5, and 9. This may be because optimal heavy metal removal occurs at pH 7, which is consistent with the findings of Gudipaty et al. (2012). Alkaline and acidic pH values decrease heavy metal removal due to the ease of heavy metal reactions with H^+^ atthese pH levels, creating competition between H^+^ and the active site on the biosorbent (Vásquez et al., 2007). Changing in temperature will affect numerous factors that are critical to heavy metal removal (Ashokkumar et al., 2017). Temperature plays a significant role in the adsorption of heavy metals. Increasing temperature increases the rate of adsorbate diffusion across the external boundary layer, leading to enhanced adsorption. Additionally, the solubility of heavy metals increases with temperature, making them more bioavailable (Bandowe et al., 2014). However, within a suitable range, rising temperature also stimulates the activity of microorganisms, enhancing their metabolism and enzyme activity, thereby accelerating bioremediation (Igiri et al., 2018). Consistent with our study, our study, Babai and Ron (1998) found that fusing an *Escherichia coli* gene responsive to heavy metals with β-galactosidase did not increase β-galactosidase expression following a temperature shift to 42°C for 30 min.

Highly significant positive correlations were observed between gene expressions and increasing in heavy metal concentrations. The results suggest that gene expression was induced by the increase in heavy metal concentrations. The fold change reached two-fold with the high concentrations in contrast to the low concentrations. Additionally, when the three heavy metals were combined, the fold change increased by 1.5–2 times for each gene compared to its expression in individual experiments. In conclusion, *E. kobei* strain FACU6 exhibited significant levels of heavy metal tolerance that could be used in bioremediation applications. The ability to survive in many ecological niches was demonstrated by the existence of distinct metal transport/resistant genes and plant growth-promoting genes. However, further research is needed to fully understand these processes and optimize them for practical applications.

## Data Availability

The original contributions presented in the study are publicly available. This data can be found here: https://www.ncbi.nlm.nih.gov/search/all/?term=PRJNA858462.
