# Supplementary material for: Draft genome analysis for Enterobacter kobei, a promising lead bioremediation bacterium
Source: Front Bioeng Biotechnol. 2024 Jan 8;11:1335854. doi: 10.3389/fbioe.2023.1335854 (PMC10800491; doi:10.3389/fbioe.2023.1335854)
Supplement: Supplementary file 1 [file DataSheet1.docx]

Supplementary Material

# Supplementary Figures:


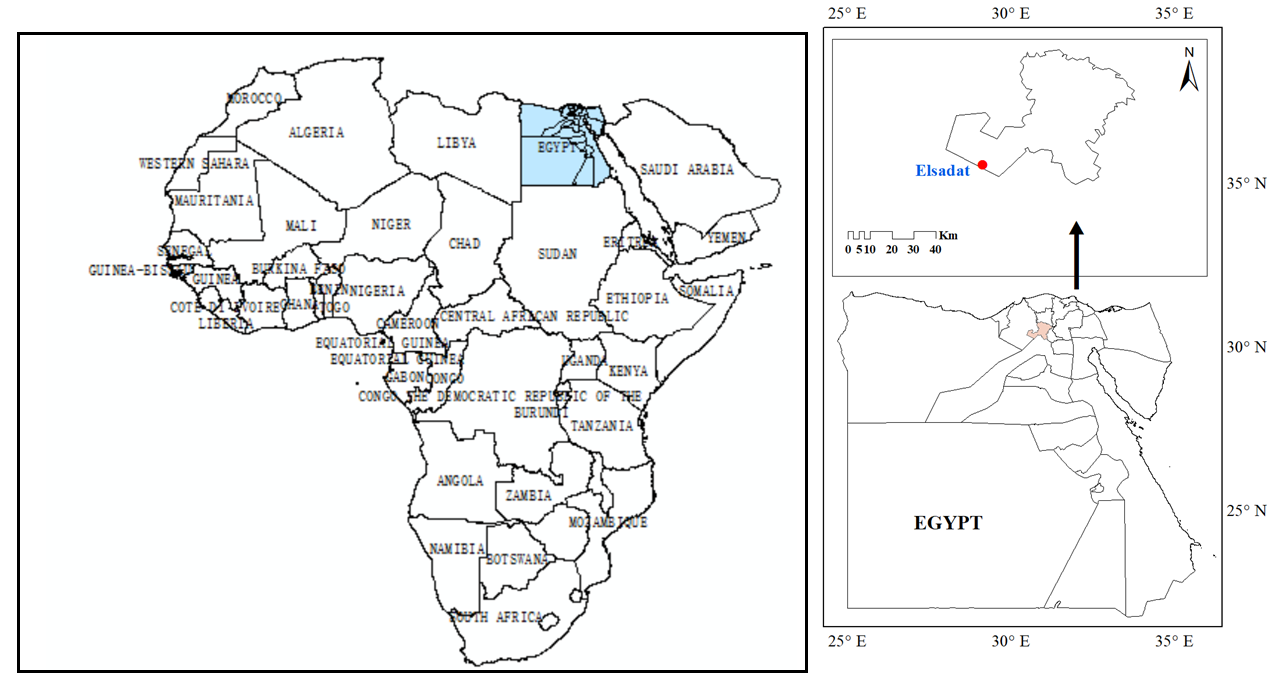


**Supplementary Figure 1.** The sample collection location from Elsadat city, Egypt


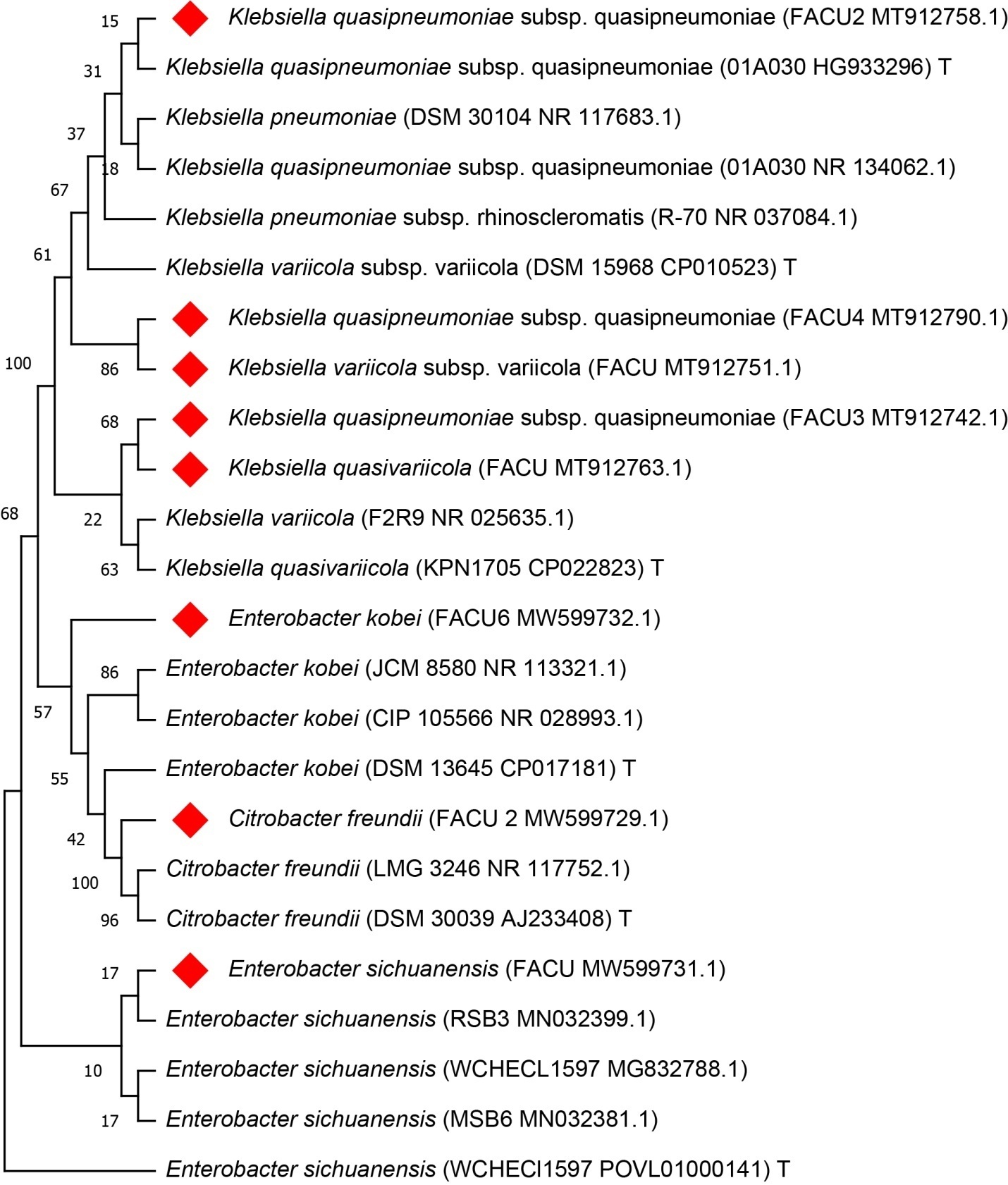


**Supplementary Figure 2.** A Maximum likelihood (ML) phylogenetic tree of eight lead-resistant strains, based on *16S rRNA* gene sequence. Bootstrapping was performed for the tree with 1000 replicates. Phylogenetic analyses were conducted in MEGA 11.


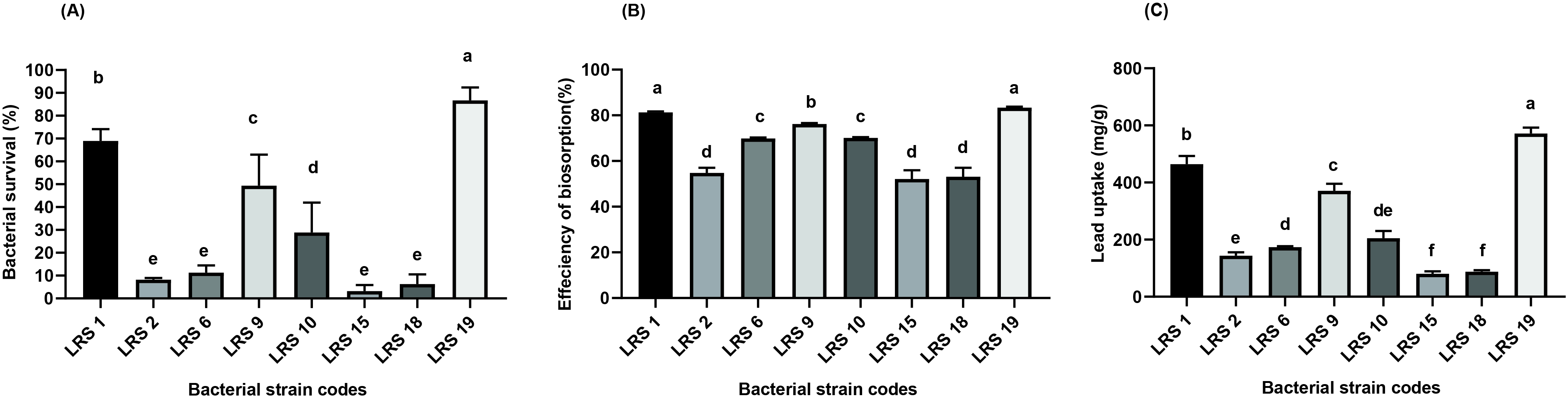


Supplementary Figure 3. Evaluating the ability of the isolated bacterial strains to resist lead through determination of bacterial survival (%) on lead stress

# Supplementary Tables

Supplementary Table 1 The primers name, primers sequences and the expected amplicon size

| **Primer name** | **Orientation 5ʹ to 3ʹ** | **Amplicon size (bp)** | **References** |
| --- | --- | --- | --- |
| *27 F* | AGAGTTTGATCMTGGCTCAG | 1500 | (Reysenbach et al. 1992; Elarabi et al. 2020) |
| *1492 R* | TACGGCTACCTTGTTACGACTT |  |  |
| *CadA -F* | GTGAATGGGCAGAGGCATCT | 180 | This study |
| *CadA -R* | CCACAGCGATATCGTCCACA |  |  |
| *pbrC -F* | GCCAAAGAACCATGCCAGTG | 163 | This study |
| *pbrC -R* | ATATGGGTGCCAGCTTGCTT |  |  |
| *arsB -F* | GGTCTATGCGCT GGAGCA ATTGAA | 500 | This study |
| *arsB -R* | TGCTGGGCATGTTGTTCATTACCG |  |  |
| *arsC -F* | GCAGCATTCTTTCCGAAGCCATGT | 215 | This study |
| *arsC -R* | TCGCAA ACG GTG ATGACGATG T |  |  |
| *U16SRT-F* | ACTCCTACGGGAGGCAGCAGT | 180 | (Clifford et al. 2012) |
| *U16SRT-R* | TATTACCGCGGCTGCTGGC |  |  |
| *U gyrB-F* | CGATTGTGTCCGTTAAAGTGC | 140 | (Burghardt and Droleskey 2006) |
| *U gyrB-R* | TGCAAACGCTCACCAACTG |  |  |

Supplementary Table 2 The physicochemical analysis and heavy metal contents of collected samples

| **Locations** | **pH** | **EC**  **(mS/cm)** | **Heavy metal contents in wastewater samples (mg/L)** | | | | | | | | | | | | | | | | | |  |
| --- | --- | --- | --- | --- | --- | --- | --- | --- | --- | --- | --- | --- | --- | --- | --- | --- | --- | --- | --- | --- | --- |
|  |  |  | **As** | | **Cd** | | **Cr** | | **Cu** | | **Fe** | | **Mn** | | **Ni** | | **Pb** | | **Zn** | |  |
| Sample no. 1 | 5.6 ± 0.1 | 1.12  ±  0.06 | | 0.05 ± 0.01 | | 0.05 ± 0.01 | | 0.5  ± 0.01 | | 0.24 ± 0.1 | | 25.7  ±  0.1 | | 0.87± 0.06 | | 0.01±  0.1 | | 0.08  ± 0.01 | | 0.3  ± 0.1 | |
| Sample no. 2 | 6  ± 0.2 | 1.12  ±  0.04 | | 0.001 ±  0 | | 0.001 ±  0 | | 0.001  ±  0 | | 0.15 ± 0.01 | | 2.1  ±  0.1 | | 0.05± 0.01 | | 0.001  ±  0 | | 0.09  ± 0.01 | | 0.18± 0.01 | |
| The US EPA standard |  |  | | 0.01 | | 0.005 | | 0.05 | | 1.3 | | 0.3 | | 0.05 | | 0.02 | | 0.01 | | 5.00 | |
| These physicochemical analysis and heavy metal contents of industrial wastewater samples were analyzed in triplicates and the results were described as mean with standard deviations (±SD). | | | | | | | | | | | | | | | | | | | | | |

Supplementary Table 3 The MIC of the twenty bacterial isolates

| **Isolates code** | **MTC (mg/L)** | **Isolates code** | **MTC (mg/L)** |
| --- | --- | --- | --- |
| LRS 1 | **2800** | LRS 11 | 1200 |
| LRS 2 | **2800** | LRS 12 | 1400 |
| LRS 3 | 1200 | LRS 13 | 1200 |
| LRS 4 | 1400 | LRS 14 | 1200 |
| LRS 5 | 1200 | LRS 15 | **2800** |
| LRS 6 | **2800** | LRS 16 | 1400 |
| LRS 7 | 1200 | LRS 17 | 1400 |
| LRS 8 | 1600 | LRS 18 | **2800** |
| LRS 9 | **2800** | LRS 19 | **3000** |
| LRS 10 | **2800** | LRS 20 | 1600 |

Supplementary Table 4 Analysis of FACU6 resistome

| **RGI Criteria** | **ARO Term** | **SNP** | **Detection Criteria** | **AMR Gene Family** | **Drug class** | **Resistance mechanism** | **% Identity of Matching Region** | | **% Length of Reference Sequence** |
| --- | --- | --- | --- | --- | --- | --- | --- | --- | --- |
| Perfect | *ACT-28* |  | protein homolog model | ACT beta-lactamase | carbapenem, cephalosporin, cephamycin, penam | antibiotic inactivation | 100 | 100 | |
| Strict | *FosA2* |  | protein homolog model | fosfomycinthioltransferase | phosphonic acid antibiotic | antibiotic inactivation | 95.04 | 100 | |
| Strict | *marA* |  | protein homolog model | resistance-nodulation-cell division (RND) antibiotic efflux pump, General Bacterial Porin with reduced permeability to beta-lactams | fluoroquinolone antibiotic, monobactam, carbapenem, cephalosporin, glycylcycline, cephamycin, penam, tetracycline antibiotic, rifamycin antibiotic, phenicol antibiotic, penem, disinfecting agents and antiseptics | antibiotic efflux, reduced permeability to antibiotic | 93.55 | 99.21 | |
| Strict | *emrR* |  | protein homolog model | major facilitator superfamily (MFS) antibiotic efflux pump | fluoroquinolone antibiotic | antibiotic efflux | 93.14 | 100 | |
| Strict | *emrB* |  | protein homolog model | major facilitator superfamily (MFS) antibiotic efflux pump | fluoroquinolone antibiotic | antibiotic efflux | 92.43 | 100.59 | |
| Strict | *rsmA* |  | protein homolog model | resistance-nodulation-cell division (RND) antibiotic efflux pump | fluoroquinolone antibiotic, diaminopyrimidine antibiotic, phenicol antibiotic | antibiotic efflux | 85.25 | 100 | |
| Strict | *adeF* |  | protein homolog model | resistance-nodulation-cell division (RND) antibiotic efflux pump | fluoroquinolone antibiotic, tetracycline antibiotic | antibiotic efflux | 41.36 | 97.92 | |
| Strict | *H-NS* |  | protein homolog model | major facilitator superfamily (MFS) antibiotic efflux pump, resistance-nodulation-cell division (RND) antibiotic efflux pump | macrolide antibiotic, fluoroquinolone antibiotic, cephalosporin, cephamycin, penam, tetracycline antibiotic | antibiotic efflux | 95.59 | 100 | |
| Strict | *msbA* |  | protein homolog model | ATP-binding cassette (ABC) antibiotic efflux pump | nitroimidazole antibiotic | antibiotic efflux | 94.67 | 100 | |
| Strict | *ramA* |  | protein homolog model | resistance-nodulation-cell division (RND) antibiotic efflux pump, General Bacterial Porin with reduced permeability to beta-lactams | fluoroquinolone antibiotic, monobactam, carbapenem, cephalosporin, glycylcycline, cephamycin, penam, tetracycline antibiotic, rifamycin antibiotic, phenicol antibiotic, penem, disinfecting agents and antiseptics | antibiotic efflux, reduced permeability to antibiotic | 92.74 | 100 | |
| Strict | *adeF* |  | protein homolog model | resistance-nodulation-cell division (RND) antibiotic efflux pump | fluoroquinolone antibiotic, tetracycline antibiotic | antibiotic efflux | 60.77 | 99.15 | |
| Strict | *oqxA* |  | protein homolog model | resistance-nodulation-cell division (RND) antibiotic efflux pump | fluoroquinolone antibiotic, glycylcycline, tetracycline antibiotic, diaminopyrimidine antibiotic, nitrofuran antibiotic | antibiotic efflux | 90.54 | 100 | |
| Strict | *KlebsiellapneumoniaKpnF* | | protein homolog model | major facilitator superfamily (MFS) antibiotic efflux pump | macrolide antibiotic, aminoglycoside antibiotic, cephalosporin, tetracycline antibiotic, peptide antibiotic, rifamycin antibiotic, disinfecting agents and antiseptics | antibiotic efflux | 87.16 | 100 | |
| Strict | *Klebsiella pneumonia KpnE* | | protein homolog model | major facilitator superfamily (MFS) antibiotic efflux pump | macrolide antibiotic, aminoglycoside antibiotic, cephalosporin, tetracycline antibiotic, peptide antibiotic, rifamycin antibiotic, disinfecting agents and antiseptics | antibiotic efflux | 82.5 | 100 | |
| Strict | *baeR* |  | protein homolog model | resistance-nodulation-cell division (RND) antibiotic efflux pump | aminoglycoside antibiotic, aminocoumarin antibiotic | antibiotic efflux | 95.4 | 100 | |
| Strict | *vanG* |  | protein homolog model | glycopeptide resistance gene cluster, Van ligase | glycopeptide antibiotic | antibiotic target alteration | 39.09 | 104.87 | |
| Strict | *CRP* |  | protein homolog model | resistance-nodulation-cell division (RND) antibiotic efflux pump | macrolide antibiotic, fluoroquinolone antibiotic, penam | antibiotic efflux | 99.05 | 100 | |
| Strict | *Haemophilus influenzae* PBP3 conferring resistance to beta-lactam antibiotics | *D350N*, *S357N* | protein variant model | Penicillin-binding protein mutations conferring resistance to beta-lactam antibiotics | cephalosporin, cephamycin, penam | antibiotic target alteration | 53.1 | 96.39 | |
| Strict | *Escherichia coliUhpT* with mutation conferring resistance to fosfomycin | *E350Q* | protein variant model | antibiotic-resistant UhpT | phosphonic acid antibiotic | antibiotic target alteration | 93.95 | 100 | |
| Strict | *Escherichia coli AcrAB-TolC* with *MarR* mutations conferring resistance to ciprofloxacin and tetracycline | | protein overexpression model | resistance-nodulation-cell division (RND) antibiotic efflux pump | fluoroquinolone antibiotic, cephalosporin, glycylcycline, penam, tetracycline antibiotic, rifamycin antibiotic, phenicol antibiotic, disinfecting agents and antiseptics | antibiotic target alteration, antibiotic efflux | 89.58 | 100 | |

# Supplementary material and methods

**Genome analysis**

Raw data quality control was assessed using the fastp v0.12.4 tool (Chen et al., 2018). Following that, SPAdes v3.13.1 was used to de novo assemble the filtered reads (Bankevich et al., 2012). The estimation of the assembly files was performed utilizing QUAST [v5.2.0](https://downloads.sourceforge.net/project/quast/quast-5.2.0.tar.gz)(Gurevich et al., 2013). Contigs gene annotation were performed utilizing three different tools (Bakta v1.5.1 (Schwengers et al., 2021), Prokka v 1.14.6 (Seemann, 2014) and RAST (Aziz et al., 2008)) and gene count was visualized using UniprotR (<https://pubmed.ncbi.nlm.nih.gov/31843688/>). The data were also subsequently analyzed by PATRIC's genome analysis service (Wattam et al., 2017). Using BWA v 0.7.17-r1188, previously filtered reads were mapped to the reference (Li and Durbin, 2009). BCFtools v 1.9 and SAMtools v 1.7 were used to accomplish variant detection and filtration (Li, 2011). When compared to protein families identified from PATtyFams, BLAST v 2.12.0+ was utilized to detect the most similar sequences. Multiple sequence alignment was carried out utilizing MAFFT v7.505 (Katoh et al. 2002), followed by the creation of a maximum likelihood phylogenetic tree with 1000 bootstrap replications using IQ-TREE v1.6.12 (Nguyen et al., 2015). Calculations using the GTR1F1I1G4 fit model were made. iTol was used to visualize the phylogenetic tree (Letunic and Bork, 2021). The assembled genome was examined for plasmids using PLATON v1.6-1 (Schwengers et al., 2020), PlasmidFinder v2.1.6 (Carattoli et al., 2014) and Abricate v 0.8.13 (https://github.com/tseemann/abricate). The genome annotation service provided by PATRIC used a k-mer-based Antimicrobial Resistant (AMR) gene detection method to assign functional annotation to widespread antibiotic resistance mechanisms (Wattam et al., 2017). The Comprehensive Antibiotic Resistance Database (CARD)'s Gene Identifier (RGI) v5.1.1 tool was used to examine the bacterial resistome; incomplete genes were removed and contigs>20,000 bp were used to make predictions (McArthur et al., 2013).
